# O3.7. EFFECT OF N-ACETYLCYSTEINE ON BRAIN GLUTAMATE LEVELS AND RESTING PERFUSION IN SCHIZOPHRENIA

**DOI:** 10.1093/schbul/sby015.205

**Published:** 2018-04-01

**Authors:** Grant McQueen, Alice Egerton, John Lally, Fernando Zelaya, David J Lythgoe, Gareth J Barker, James Stone, Philip McGuire, James MacCabe, Tracey Collier

**Affiliations:** Institute of Psychiatry, Psychology & Neuroscience, King’s College London

## Abstract

**Background:**

Schizophrenia may be associated with elevations in glutamate levels in the anterior cingulate cortex (ACC), and this may be particularly apparent in patients who have not responded well to conventional antipsychotic treatment (Egerton et al., 2012; Mouchliantis et al., 2016). This suggests that compounds that can decrease ACC glutamate levels may have therapeutic potential for this group. N-acetylcysteine (NAC) is one such compound, currently under investigation as an adjunctive therapy for schizophrenia. The effects of NAC on brain glutamate levels and physiology in schizophrenia have not been previously evaluated. The primary aim of this study was to examine whether a single oral dose of NAC can alter brain glutamate levels in schizophrenia. The secondary aim was to characterise the effects of NAC on regional brain perfusion.

**Methods:**

In a double-blind placebo-controlled crossover study, twenty patients with a diagnosis of schizophrenia underwent two 3 Tesla MRI scans, performed one week apart, and following administration of a single oral dose of 2400mg NAC or matching placebo. Proton magnetic resonance spectroscopy (1H-MRS) was used to investigate the effect of NAC on glutamate and Glx (glutamate plus glutamine) levels scaled to creatine (Cr) in the anterior cingulate cortex (ACC) and in the right caudate nucleus. Pulsed continuous arterial spin labelling (pCASL) was used to measure the effects of NAC on resting cerebral blood flow (CBF) in the same regions. 1H-MRS spectra were analysed using LCModel version 6.3-0I using a standard basis set. Individual CBF maps were pre-processed in the Automatic Software for ASL Processing (ASAP) toolbox running in SPM-8 in Matlab 6.5. The effects of NAC on 1H-MRS metabolite levels were determined using paired samples t-tests. Changes in rCBF were determined using within-subjects, second-level analysis implemented in SPM-8.

**Results:**

In the ACC, Glx/Cr was significantly reduced in the NAC compared to placebo condition (t(17) = 2.40; P = .03, d = 0.64). There was no significant effect of condition on Glu/Cr in the ACC, or on Glx/Cr or Glu/Cr in the right caudate nucleus, or on any of the other metabolites quantifiable from the 1H-MRS spectra. There were no significant differences in CBF in the ACC (mean (SD) placebo = 47.22 (8.81); NAC = 46.83 (7.29); t(18) = .349, P = .73) or in the right caudate nucleus (mean (SD) placebo = 37.51 (7.48); NAC = 37.77 (6.71); t(18) -.310, P = .76) in the NAC compared to placebo condition. There was also no significant difference in global CBF between conditions (mean (SD) placebo = 39.64 (10.02); NAC = 40.03 (9.13); t(18) = -.398, P = .70).

**Discussion:**

These results provide preliminary evidence that NAC may reduce ACC glutamate metabolites in schizophrenia. Future studies will need to determine the extent to which reductions in glutamate metabolites following a single dose of a glutamatergic compound are indicative of longer-term efficacy in improving symptoms.

